# Accurate prediction of electricity consumption using a hybrid CNN-LSTM model based on multivariable data

**DOI:** 10.1371/journal.pone.0278071

**Published:** 2022-11-23

**Authors:** Jaewon Chung, Beakcheol Jang

**Affiliations:** 1 Graduate School of International Studies, Yonsei University, Seoul, South Korea; 2 Graduate School of Information, Yonsei University, Seoul, South Korea; J.C. Bose University of Science and Technology, YMCA, INDIA, INDIA

## Abstract

The stress placed on global power supply systems by the growing demand for electricity has been steadily increasing in recent years. Thus, accurate forecasting of energy demand and consumption is essential to maintain the lifestyle and economic standards of nations sustainably. However, multiple factors, including climate change, affect the energy demands of local, national, and global power grids. Therefore, effective analysis of multivariable data is required for the accurate estimation of energy demand and consumption. In this context, some studies have suggested that LSTM and CNN models can be used to model electricity demand accurately. However, existing works have utilized training based on either electricity loads and weather observations or national metrics e.g., gross domestic product, imports, and exports. This binary segregation has degraded forecasting performance. To resolve this shortcoming, we propose a CNN-LSTM model based on a multivariable augmentation approach. Based on previous studies, we adopt 1D convolution and pooling to extract undiscovered features from temporal sequences. LSTM outperforms RNN on vanishing gradient problems while retaining its benefits regarding time-series variables. The proposed model exhibits near-perfect forecasting of electricity consumption, outperforming existing models. Further, state-level analysis and training are performed, demonstrating the utility of the proposed methodology in forecasting regional energy consumption. The proposed model outperforms other models in most areas.

## Introduction

### Literature review

Electricity is both a primary index representing the economic welfare of a country and the primary gear of modern convenience [1, 2]. Power outages threaten the quality of life and the economy of a state [3] and power shortages damage the economic growth of emerging nations [3, 4]. Even nations with well-developed power systems may experience sudden power failures [3]. Climate change and adverse weather conditions such as heatwaves, tornadoes, and heavy snowfall increase the chances of electrical failure. Therefore, the accurate forecasting of electricity consumption has become more important to ensure a stable electricity supply.

Several studies have been conducted to predict energy consumption trends. Long short-term memory (LSTM) models combined with convolutional neural networks (CNNs) have been proposed for the hourly and daily forecast of energy demands, which outperform multilayer perceptron (MLP) and recurrent neural network (RNN) models [5–7]. Electricity load forecasting (LF) studies [5, 7, 8] have utilized historical electricity load and climate data uploaded in real time. Most energy consumption forecasting studies [9–12] have been conducted considering factors such as population, import/export values, and gross domestic product (GDP). The nomenclature utilized in this study is presented in Table 1.

**Table 1:** The nomenclature.

Nomenclature	
*CNN*	Convolutional Neural Network
*DL*	Deep Learning
*EEC*	Electrical Energy Consumption
*GDP*	Gross Domestic Product
*LF*	Load Forecasting
*LSTM*	Long-Short Term Memory
*MAPE*	Mean Absolute Percentage Error
*MLP*	Multilayer Perceptron
*MTLF*	Mid-term Load Forecasting
*RMSE*	Root Mean Squared Error
*RNN*	Recurrent Neural Network
*ReLU*	Rectified Linear Units
*STLF*	Short-term Load Forecasting
*TEC*	Total Energy Consumption

Most existing studies on electrical energy consumption (EEC) and total energy consumption (TEC) forecasting methods focus on macroeconomic features, including population, GDP, and import/export values. On the other hand, existing studies on short-term load forecasting (STLF) and mid-term load forecasting (MTLF) consider only historical load and weather/time information [13]. However, this binary separation of components constrains the forecasting accuracy of energy demand and consumption—the components must be integrated to improve prediction performance.

Table 2 presents an overview of existing load and energy consumption forecasting, including their input and target variables, forecasting time intervals, and forecasting areas. In terms of forecasting time intervals, energy forecasting can be categorized into two groups: 1) monthly, seasonal, and annual forecasting [1, 10–12, 14–16] and 2) per-minute, hourly, and daily forecasting [5–8, 17]. The former type considers socio-economic input variables from GDP, population, sales index, production index, import/export, demographic, personality, and Google Trends data. The latter type involves observations of the actual electric load, voltage, submetering, and weather. Further, the target areas of the two types are different. Methods of the first group predict energy consumption at state [9, 14, 15], national [11, 12, 14, 16], and global [10] levels whereas those in the second group [6–8, 17] predict the energy consumption of relatively smaller areas. In [9], a neural network ensemble approach based on a novel sparse adaboost framework and an echo state network was used to improve generalization ability and construct nonlinear relations between electricity demand and other factors. The proposed model was validated by using it to predict the industrial electricity consumption of Hubei Province of China. However, further real-life applications in different regions of China are required to establish its generality. In [10], Google Trends data, including the search history of certain keywords, were used as the input dataset and an online big data-driven oil consumption forecasting approach was constructed for application alongside statistical and machine learning (ML) techniques. It was the first attempt to use the trends of google search data to predict uncertain yet essential oil consumption. It also proved the predictive power of Google search trends through relationship investigation. However, the proposed prediction improvement techniques did not explore deep learning (DL) modules. In [14], the forecasting speed of residential electricity consumption was prioritized, contrary to most residential electricity consumption studies that focus on forecasting accuracy. The authors proposed a hybrid Improved Whale Optimization Algorithm—Optimized Grey Seasonal Variation Index which exhibited both high accuracy and fast convergence. Consideration of forecasting speed is practical because real-life implementation requires fast forecasting. The aforementioned model also exhibited “excellent” forecasting accuracy. In [8], the periodic part of the household residual load forecasting was modeled based on the behavioral patterns of overestimated/underestimated residual components. The model exhibited a significant improvement in the prediction accuracy of periodic residual demand. When combined with climatic data, it also improved total power consumption prediction. Nevertheless, as the real dataset of the experiment pertained to a single household, its application in broader residential households remains to be established. To improve the performance of RNN on electric load forecasting at a specific time, a recurrent inception convolution neural network combining RNN and 1D CNN was proposed in [6]. To this end, a 1D CNN inception model was used to balance prediction time and hidden state vector values. Its performance was verified based on power usage data obtained from three distribution complexes in South Korea. The model outperformed the benchmarks of MLP, RNN, and 1D CNN. However, because there are multitudinous power distribution complexes in South Korea, its application to the entire country requires further verification.

**Table 2:** Some typical types of energy forecasting techniques.

Ref.	Input Variables	Output variables	Prediction Interval	Prediction Area
[9]	Industrial sales, producer price index, export delivery value, export value, import value, etc.	Industrial electricity consumption	Monthly	Hubei, China
[10]	Google trend data	Oil consumption	Monthly	World
[11]	GDP, population, export value, import value	EEC/TEC	Annual	China
[14]	Seasonal residential electricity consumption history	EEC, Residential electricity consumption	Seasonal	USA, 4 regions of China
[15]	Electricity consumption history	EEC	Monthly	Bejaia, Algeria
[12]	Gross electricity generation, GDP, population, installed capacity, import value, export value, total subscribership	EEC	Annual	Turkey
[16]	Energy consumption history, moving average, bias, momentum, rate of change, etc.	TEC	Monthly	USA
[8]	Electricity load history, climatic data	Residential electricity load	Hourly	An individual household in Montreal, Canada
[17]	Active power, reactive power, voltage, global intensity, etc	Residential electricity consumption	15 minutes	An individual residential building in Paris, France
[7]	Global active power, global reactive power, voltage, global intensity, sub metering	Residential power consumption	Hourly	An individual household in France
[5]	Electricity load history (ISO-NE, NYISO 13 years of electricity load of New England, New York)	Electricity load	Hourly	New England and New York, USA
[6]	Electricity load history, climatic data, time information, electricity rates, number of sensors	Electricity load	Daily	Three distribution complexes of South Korea

### Research gap and motivation

In this paper, we present a novel and accurate hybrid CNN-LSTM model that uses multivariable data to forecast EEC. Further, we explain the potential problems and roles of various state-level forecasting components, such as regional import and export values. The proposed technique utilizes broad and necessary components of state-level time-series forecasting techniques. The proposed technique uses LSTM layers to process and predict based on time-series data, and the output depends not only on the current input but also on the inputs from the past. The hybrid CNN-LSTM also utilizes convolutional and pooling layers to extract various aspects and objects in a time series, and achieves differentiation at a relatively low computational cost. Moreover, it uses a multivariable dataset containing the data of electric load, export values, and import values to estimate the electricity usage of the following month. Industrial electricity usage and imports/exports are directly proportional to each other; hence, the value of international trade represents the present and future costs of electricity closely. Our technique also allows users to analyze and predict electricity consumption of any region, in addition to that on a national level.

South Korea was deemed to be an adequate data source to identify the correlation between import/export values, industrial electricity usage, and total electricity consumption. As an advocate for climatic environmentalism, the South Korean government has recently reduced the number of nuclear and thermal power plants in the country. This endeavor has led to nationwide concerns regarding electricity shortage, as 72.9% of the GDP [18] of the country relies on importing/exporting sectors of the manufacturing industry, which require more than one-third of the nation’s total EEC [19]. Moreover, manufacturing and production firms in South Korea are primarily distributed in specific regions, necessitating accurate state-level energy forecasting for smooth operation. Therefore, 16 regions in South Korea are analyzed to train a novel nationwide DL-based EEC forecasting model in this paper.

In this study, the Open Data Portal [20], Open MET Data Portal [21], and Trade Statistics [22] government databases of South Korea are used, with each offering monthly data on energy consumption, import/ export values, and temperature and rainfall data during the period between January 2004 and December 2020. Our augmented multivariable CNN-LSTM is observed to exhibit a root mean square error (RMSE) of 0.165 in the nation-level experiment, outperforming conventional deep learning-based energy forecasting algorithms—the RMSEs of MLP, RNN, and LSTM are 0.4521, 0.1713, and 0.174, respectively. Moreover, in regional forecasting, the CNN-LSTM model using the proposed data processing method outperforms the alternatives in all the areas, accurately predicting 16 states in terms of RMSE and mean absolute percentage error (MAPE).

### Contribution

As mentioned in the Literature Review and the Research Gap and Motivation sections, previous approaches to the prediction of electricity consumption and demands have not considered the two types of input factors simultaneously. However, greater integrity in input is necessary to improve prediction accuracy. Moreover, monthly state-level predictions are required to account for variable energy consumption patterns of different states. Finally, we utilize an interpolation technique for convergence between monthly data points. While the time units of LF datasets are as detailed as seconds and milliseconds, the finest unit of time in the case of EEC/TEC is a month. Our interpolation bridges these gaps between different data points and ensures better prediction scores. The various contributions of this paper are as follows:

- We present a novel EEC forecasting method based on small datasets using data augmentation.
- The proposed model integrates input components of EEC/TEC forecasting and LF to improve prediction performance.
- To the best of our knowledge, this is the first study to conduct the state-level monthly EEC forecasting for South Korea.

### Paper organization

The rest of this paper is structured as follows. In Materials and Methods, the proposed deep learning model is introduced along with the requisite background information. Experimental validation of the proposed method is presented in the Results section. In the Discussion section, the motivation of the study is re-stated and the results obtained using the proposed method are analyzed. Finally, the study is concluded in the Conclusions section.

## Materials and methods

The proposed energy-consumption forecasting model comprises three steps. In the first step, the univariable and multivariable time series data based on continuous observations of climatological/economic information are analyzed and a collective dataset is constructed. In the second step, a resampling method and spline interpolation are applied to generate the simulation dataset comprising continuous polynomial segments. An overview of deep learning technology for sequential data is introduced in the third step with the objective of preserving an accurate one-day and one-month forecasting horizon for electricity consumption.

### Extraction of important features

**Fig 1:**
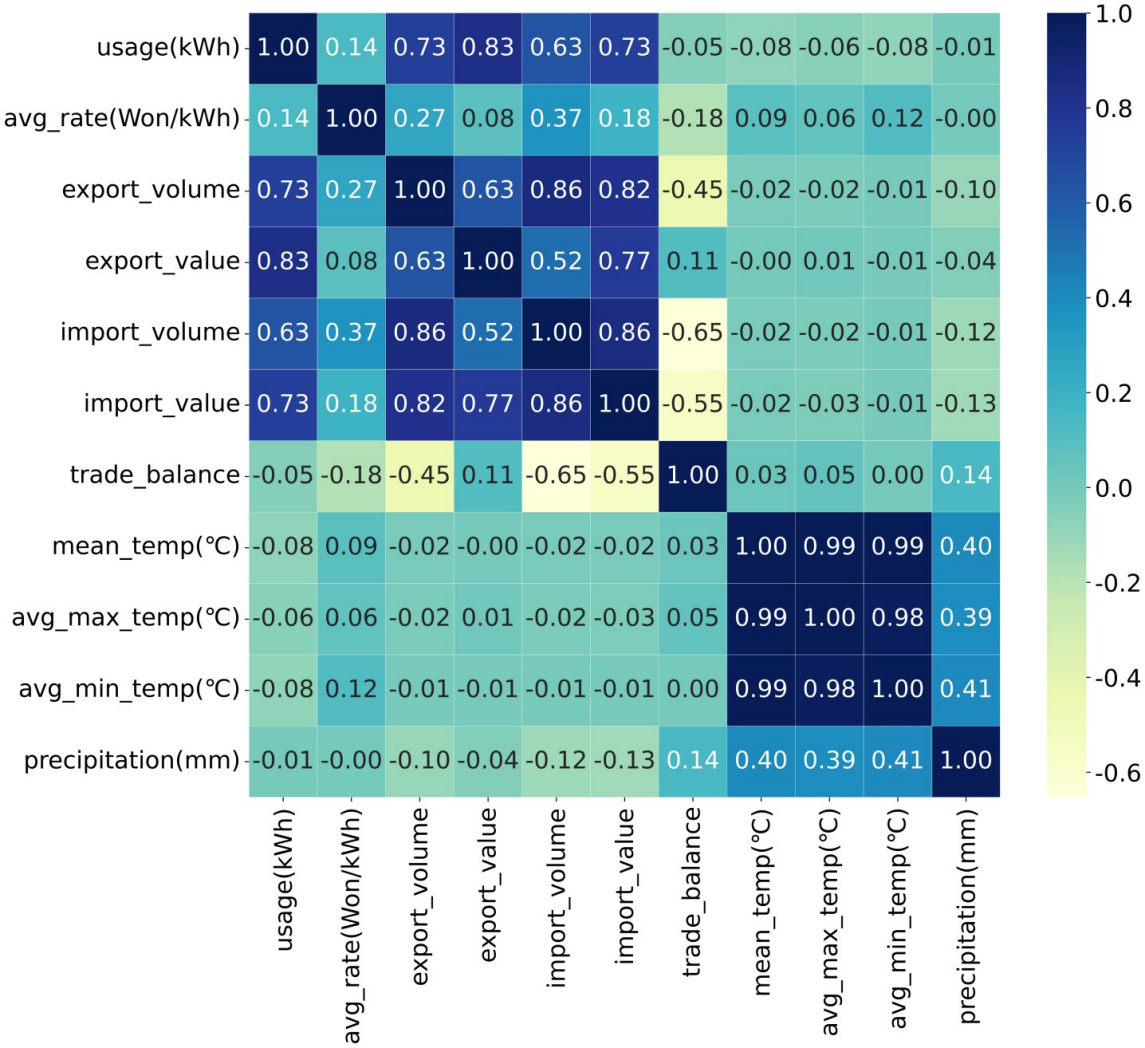
Correlation coefficients of input variables.

In [1, 2], a close correlation was reported between GDP and power consumption. However, the annuity of GDP impedes its application in deep learning, as annual GDP index data is too sparse for DL model training. To offset this challenge, monthly macroeconomic features, including import/export values, volume, and trade gains and losses from Korea Customs Service, are collected in this study. Fig 1 depicts the Pearson correlation coefficients of all 11 features. The import and export values exhibit the highest correlation with power usage. Climatic factors, such as temperature and precipitation, exhibit weak relationships with the target variable.

Regional analysis is another important sector of energy forecasting because energy consumption is correlated to the local conditions of each community. These conditions include weather, population, and economic factors. However, although studies have been conducted on provincial energy forecasting [5, 9, 14], nationwide forecasting of each region remains to be performed.

### Data augmentation

Certain input variables cannot be collected at the frequency of a target variable. Specifically, macroeconomic factors, such as import and export values, are usually collected monthly, quarterly, and annually, while empirical observations of temperature, rain, and electric load are measured every second, minute, or hour. To preserve the characteristics of both domains, a new dataset of daily frequency can be used as a middle ground.

**Fig 2:**
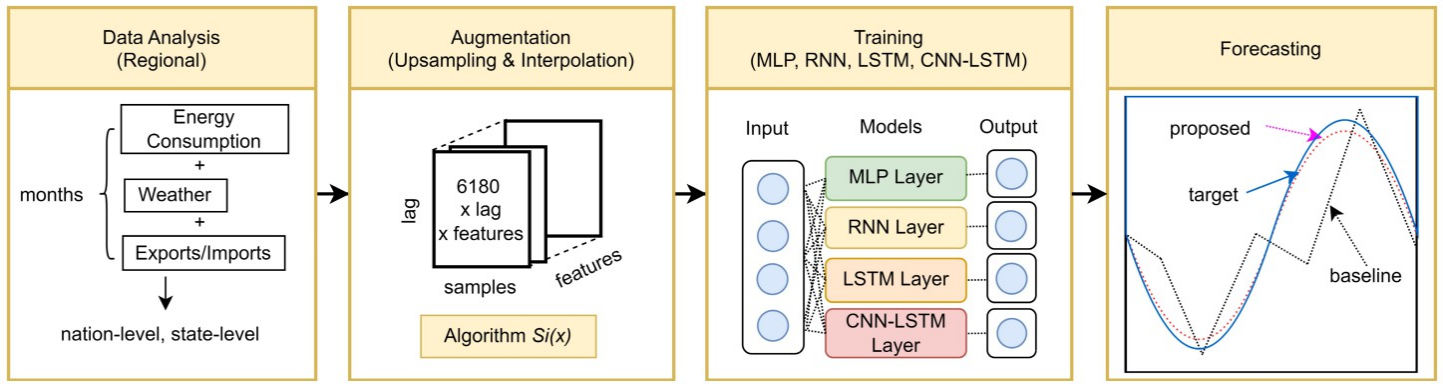
Visualization of the proposed methodology.

Fig 2 illustrates the workflow of the proposed method. Once the rows of new daily values are generated, interpolation is used to generate the simulated values. In power forecasting, as mentioned in [23], piecewise cubic polynomial interpolation represents a reasonable compromise between computational cost and flexibility. In [24] as well, piecewise cubic interpolation was preferred over other spline methods to smooth the down-sampled data, suggesting a data-driven load-forecasting method. However, in the aforementioned study, a significant performance improvement was not reported in the case of the cubic spline compare to the case of quadratic spline—instead, piecewise cubic interpolation required greater computational time during the training process than the quadratic spline. Therefore, piecewise quadratic-spline interpolation is used for data augmentation in this study.

The function, S(x), interpolates each local data point piecewise to restrict Runge’s phenomenon. Here,

$$\begin{array}{c} S_{i}\left(x\right)=a_{i}+b_{i}\left(x-x_{i}\right)+c_{i}{\left(x-x_{i}\right)}^{2}, \end{array}$$ (1)

where *i* ∈ [0, 1, …, *n*] and *x* ∈ *R*. The parameters *a*_*i*_, *b*_*i*_, and *c*_*i*_ denote quadratic polynomial coefficients. A multi-dimensional array of size 6180 × *lag* × *features* defines a new daily polynomial coefficient structure. For each region, the length of the time series is taken to be 6180, *lag* denotes the length of the shifted time-frequency for single-step forecasting, and *features* denote multivariable factors from the given datasets containing data regarding electricity consumption, weather information, and import/export indexes.

### Deep learning models

#### 1. Multilayer perceptron

MLP was introduced to address the limitations of the perceptron. By stacking one or more hidden layers between the input and output layers, MLP maps the input and output nonlinearly. First, the input layer receives an input tensor of arbitrary shape. The weight of each neuron is calculated as the data transmitted from the input to the output layer. Then, the error is minimized by backpropagating the mini-batch gradient descent (for weight update) for every epoch. Notably, the performance of the feedforward network depends on the learning rate and the effectiveness of the optimizer of the backpropagation process. Finally, the output layer of the MLP yields the desired output, e.g., the classification or prediction result. The simplest form of MLP is based on linear regression and comprises a single input and output layer with no activation functions (i.e., a linear activation function). Eq (2) presents the mathematical expression of MLP, where *w* denotes the vector of weights, *x* denotes the input tensor, *b* denotes the bias, and *σ* denotes the nonlinear activation function.

$$\begin{array}{c} y=\sigma \left(\sum_{i=1}^{n}w_{i}x_{i}+b\right)=\sigma \left(W^{T}x+b\right) \end{array}$$ (2)

Despite its nonlinearity, MLP experiences overfitting and vanishing gradient problems. Overfitted models fail to predict test data correctly, thereby diminishing their feasibility. In addition, in MLPs containing hundreds of hidden layers, the backpropagation process can diminish the gradient to 0, or close to 0, impeding further training.

#### 2. Recurrent neural networks

RNNs and RNN-based models exhibit superior performance on sequence data, such as text and time series data [25]. While feedforward neural networks remember only the current input, RNNs store information regarding the temporal order and consider previous inputs as well as the current state during decision-making. As in the case of a feedforward network, an RNN first calculates the loss function on a batch (forward propagation). Then, it updates the gradients based on the current state by calculating the state memory of earlier instances (backpropagation through time).

$$\begin{array}{c} a^{\left\langle t\right\rangle }=\text{tanh}\left(W_{ax}x^{\left\langle t\right\rangle }+W_{aa}a^{\left\langle t-1\right\rangle }+b_{a}\right) \end{array}$$ (3)

$$\begin{array}{c} {\hat{y}}^{\left\langle t\right\rangle }=softmax\left(W_{ya}a^{\left\langle t\right\rangle }+b_{y}\right) \end{array}$$ (4)

Eq (3) presents a basic RNN cell, where *x*^〈*t*〉^ denotes the current input, and *a*^〈*t*−1〉^ denotes a previous hidden state with previous information. A single RNN unit takes *x*^〈*t*〉^ and *a*^〈*t*−1〉^ as inputs and outputs *a*^〈*t*〉^ to the following RNN cell. Output *a*^〈*t*〉^ is also used to predict *y*^〈*t*〉^. However, RNNs are also prone to the vanishing gradient problem, as their cells have short-term memory.

#### 3. Long-short term memory

To counteract the vanishing gradient problem of RNNs, three gates that forget, update, and output the hidden states of current and previous instances are used in LSTM [26]. In Eq (5) the forget gate decides whether to delete or retain stored memory values of the input state. If one of the values of $\Gamma_{f}^{\langle t\rangle }$ is 0, or close to 0, the LSTM cell removes information from the corresponding component of *c*^〈*t*−1〉^. If one of the values is 1, the LSTM cell retains the information. Similarly, the update gate reflects the information from the forget gate, whereas the output gate determines the outputs to be used. All these decisions are then added to the cell state, and the memory variable is subsequently delivered to the subsequent LSTM cell with a hidden state. Thus, each LSTM-cell tracks and updates a memory variable *c*^〈*t*〉^ (i.e. a cell state) at each time step, which can be different from *a*^〈*t*〉^.

$$\begin{array}{c} \Gamma_{f}^{\left\langle t\right\rangle }=\sigma \left(W_{f}\left[a^{\left\langle t-1\right\rangle },x^{\left\langle t\right\rangle }\right]+b_{f}\right) \end{array}$$ (5)

$$\begin{array}{c} \Gamma_{u}^{\left\langle t\right\rangle }=\sigma \left(W_{u}\left[a^{\left\langle t-1\right\rangle },x^{\left\langle t\right\rangle }\right]+b_{u}\right) \end{array}$$ (6)

$$\begin{array}{c} {\hat{c}}^{\left\langle t\right\rangle }=\text{tanh}\left(W_{c}\left[a^{\left\langle t-1\right\rangle },x^{\left\langle t\right\rangle }\right]+b_{c}\right) \end{array}$$ (7)

$$\begin{array}{c} c^{\left\langle t\right\rangle }=\Gamma_{f}^{\left\langle t\right\rangle }\circ c^{\left\langle t-1\right\rangle }+\Gamma_{u}^{\left\langle t\right\rangle }\circ {\hat{c}}^{\left\langle t\right\rangle } \end{array}$$ (8)

$$\begin{array}{c} \Gamma_{o}^{\left\langle t\right\rangle }=\sigma \left(W_{o}\left[a^{\left\langle t-1\right\rangle },x^{\left\langle t\right\rangle }\right]+b_{o}\right) \end{array}$$ (9)

$$\begin{array}{c} a^{\left\langle t\right\rangle }=\Gamma_{o}^{\left\langle t\right\rangle }\circ \text{tanh}\left(c^{\left\langle t\right\rangle }\right) \end{array}$$ (10)

#### 4. Convolutional neural network and long-short-term memory

**Fig 3:**
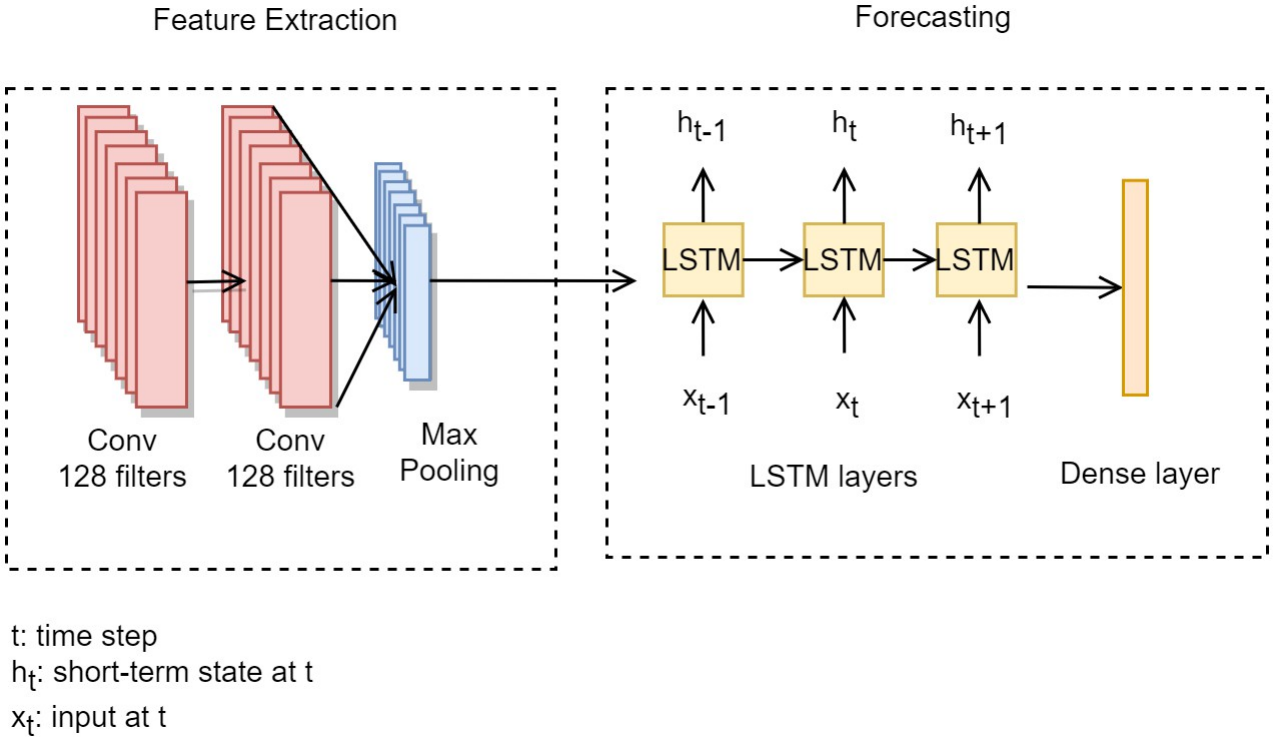
CNN-LSTM model architecture.

CNN-LSTM architectures extract detailed features from multiple variables to forecast EEC and can remember complex irregular trends [7]. The CNN-LSTM model comprises two stages—feature extraction and forecasting [27], as illustrated in Fig 3. Feature extraction comprises a 1D CNN consisting of convolution layers, an activation function, and a pooling layer. The 1D CNN extracts features from an input layer that receives multivariable macroeconomic and observational time-series sequences. Then, the CNN layer transmits the results to the LSTM layer, and the dense layer forecasts the future electricity consumption of each region.

In Eq (11), *h*_*ij*_^1^ denotes the output vector of the first convolution layer, where *i* ∈ [0, 1, …, *n*], $x_{i}^{0}=x_{1},x_{2},\ldots ,x_{n}$ denotes the input energy consumption input vector, *n* denotes the length of time-series input sequence, *j* denotes the index value of the feature map corresponding to each lag, *M* denotes the number of filters, and *W* denotes the weight vector of the kernel. $h_{ij}^{l}$ denotes the result of the *l*th convolution layer and the *i*th value of the layer. To reduce the network computation costs and the number of trainable parameters, the CNN layer uses a pooling layer to reduce the spatial size of the representation. The pooling layer $p_{ij}^{l}$ integrates a neuron filter on a previous layer into a scalar in the subsequent layer, where *T* denotes the size of the stride and *R* denotes the pooling size. Max-pooling is used in this paper, which selects the maximum value from each filter during feature extraction.

$$\begin{array}{c} h_{ij}^{1}=\sigma \left(\sum_{m=1}^{M}W_{m,j}^{1}x_{i+m-1,j}^{0}+b_{j}^{1}\right) \end{array}$$ (11)

$$\begin{array}{c} h_{ij}^{l}=\sigma \left(\sum_{m=1}^{M}W_{m,j}^{l}x_{i+m-1,j}^{0}+b_{j}^{l}\right) \end{array}$$ (12)

$$\begin{array}{c} p_{ij}^{l}=\underset{r\in R}{\text{max}}\;h_{i\times T+r,j}^{l-1} \end{array}$$ (13)

The kernel of the 1D CNN moves vertically across a feature map; hence, it receives a single integer as the filter height. In contrast, a 2D CNN has a two-dimensional kernel, e.g., a 2 × 1 filter [7]. A 1D CNN is selected in this study instead of a 2D CNN because our input data consists of a one-dimensional time series sequence. In addition, 1D CNN is more competitive in terms of computational cost than 2D CNN [28].

#### 5. Evaluation metrics

The proposed model was compared with existing ones in terms of RMSE and MAPE. RMSE calculates prediction errors and indicates how far the residuals are from the line of best fit. RMSE is useful to configure models for a specific variable and compare prediction errors between different models. RMSE is defined as follows:

$$\begin{array}{c} RMSE=\sqrt{\frac{1}{n}\sum_{t=1}^{n}{\left(C_{t}-A_{t}\right)}^{2}} \end{array}$$ (14)

where *n* denotes the number of observations, *C*_*t*_ denotes the forecasted values of consumption, and *A*_*t*_ denotes the observed actual values at time stamp *t*.

MAPE is used to compare the forecasting accuracy of different time-series models. For each forecasted point in period *t*, the prediction error is given by *e*_*t*_ = *A*_*t*_ − *C*_*t*_, and the absolute value of the percentage error, *e*_*t*_ = (*A*_*t*_ − *C*_*t*_)/*A*_*t*_, is summed and divided by the number of fitted points, *n*. MAPE corresponding to a period *t* is given by:

$$MAPE=\frac{1}{n}\sum_{t=1}^{n}|\frac{\left(A_{t}-C_{t}\right)}{A_{t}}|\times 100$$ (15)

## Results

### Data pre-processing

For data pre-processing, the multivariable time-series data are rescaled from monthly to daily periods. Each feature contains 204 rows of monthly values collected between January 2004 and December 2020 (204 months). 204 months are upsampled into 6180 days for 16 South Korean regions. The upsampled dates contain NaN values for all 3 features of historical electricity usage, export value, and import value. Historical electricity consumption is defined as the sum of residential, industrial, educational, and other electricity usages. Export and import values denote the total price of exported and imported merchandise, respectively. As mentioned in Extraction of Important Features, because the climate variable has little correlation with historical electricity usage, climatic data is excluded from experiments. The empty values are estimated using spline interpolation. The total length of the preprocessed data is taken to be 6180 × 16 × 4, without any missing values. Following previous energy forecasting studies [6], MLP is adopted as our baseline model. The baseline method is taken to be univariate single-step forecasting based on a previous consumption time series. To confirm the effect of augmentation, multivariable forecasting is also included in this experiment. The unaugmented univariate and multivariable methods are used to predict the monthly consumption based on the data of the previous two months. The proposed multivariable augmented model uses the data of the interpolated past 30 days to predict the consumption figure for each day. To compare the daily forecasts with the monthly forecasts, we extract consumption predictions for specific dates corresponding to the time intervals before interpolation.

### Model hyperparameters

To focus on the impact of data augmentation, the architecture of the DL models is simplified. The MLP consists of a single dense layer with one node and one linear activation function. Both the RNN and LSTM comprise 128 units, a ReLU (Rectified Linear Unit) activation function and a dropout layer are included following a single hidden layer. The CNN-LSTM contains 2 layers of 1D CNN with 128 filters, kernel size of 1, stride of 1, pooling size of 2, and a ReLU activation function, followed by the LSTM layer with identical hyperparameter as the LSTM-only model. All models accept a 2D array of (length of dataset, number of features) as the input and use the Adam optimizer for compilation. The final dense layer of the augmented model forecasts the power consumption for the subsequent day and those of the other predicts the consumption for the subsequent month. All models split the training and test datasets following a 70:30 ratio. The training is repeated for each model, region, and method to ensure a fair comparison.

### Comparison of national performance

**Fig 4:**
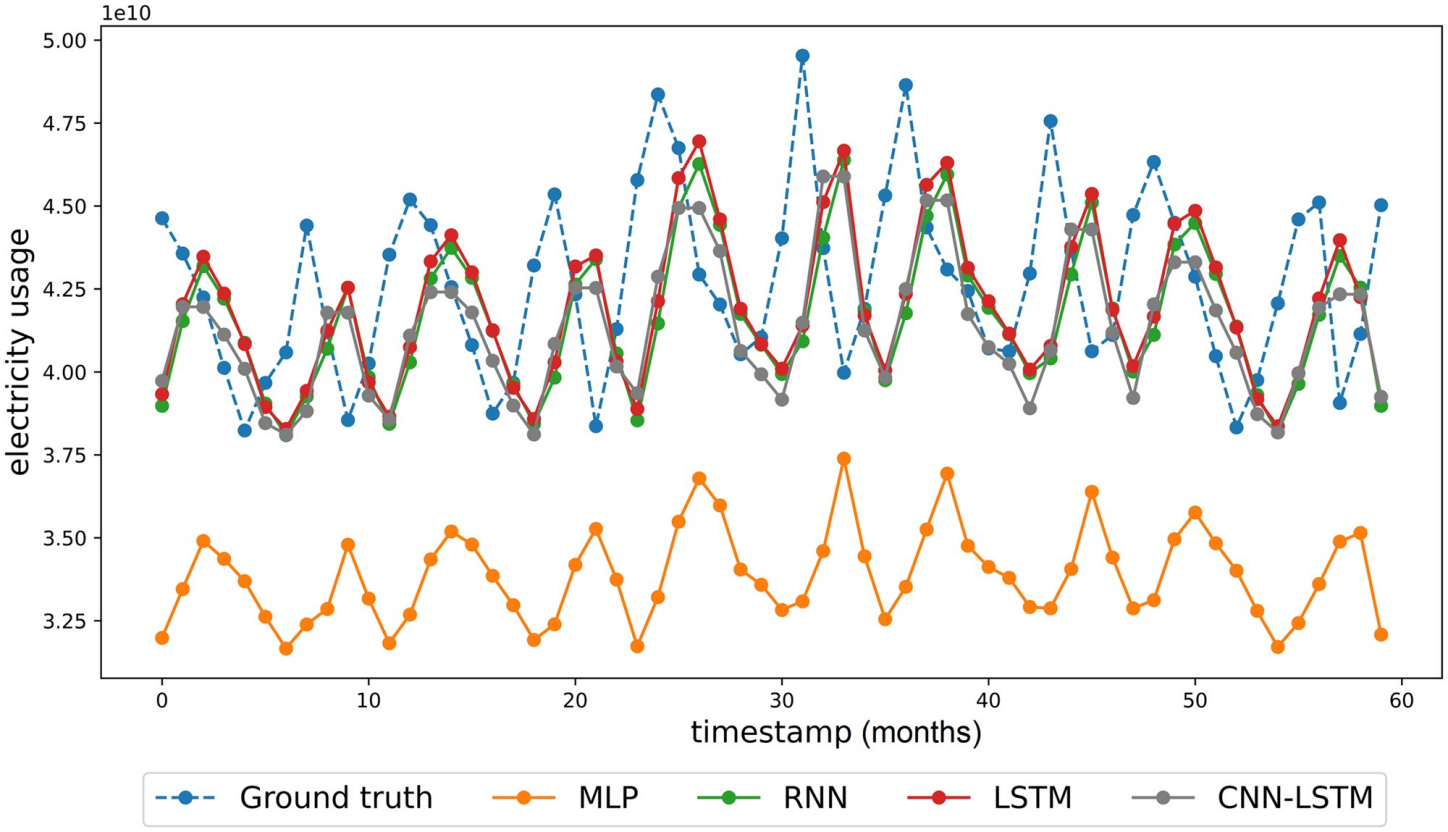
Comparison of national forecasting performance.

The performance of the proposed model is compared with those of other DL algorithms in terms of model fitness on different examples. Notably, LSTM does not always yield superior results compared to RNN [29], even though the former improves the exploding/vanishing gradient problems of RNNs. The performance of each algorithm is estimated on a basic dataset, that is univariate and without augmentation. Fig 4 compares the national electricity usage predictions obtained using deep learning models with those of the baseline monthly dataset. This figure confirms that the time-series outputs of the RNN, LSTM, and CNN-LSTM are almost identical. Table 3 summarizes the evaluation metrics used for further comparison of the models. The RMSE and MAPE scores are observed to decrease significantly with the addition of recurrent layers, and the inclusion of a 1D convolution layer is observed to improve performance additionally. Also, to further demonstrate the robustness of CNN-LSTM, we add the forecasting comparison of the USA’s electricity consumption. Retrieved from [30], the USA dataset consists of the national electricity usage and the price from January 1990 to July 2022. Same with the South Korea dataset, spline interpolation is applied to the monthly USA dataset.

**Table 3:** Forecasting performance evaluation.

**South Korea**	**MLP**	**RNN**	**LSTM**	**CNN-LSTM**
**RMSE**	0.4521	0.1798	0.174	**0.165**
**MAPE**	0.4727	0.1602	0.1589	**0.1441**
**USA**	**MLP**	**RNN**	**LSTM**	**CNN-LSTM**
**RMSE**	1.9817	0.1511	0.0889	**0.0761**
**MAPE**	1.8008	0.1068	0.0574	**0.0541**

### Regional performance comparison

**Fig 5:**
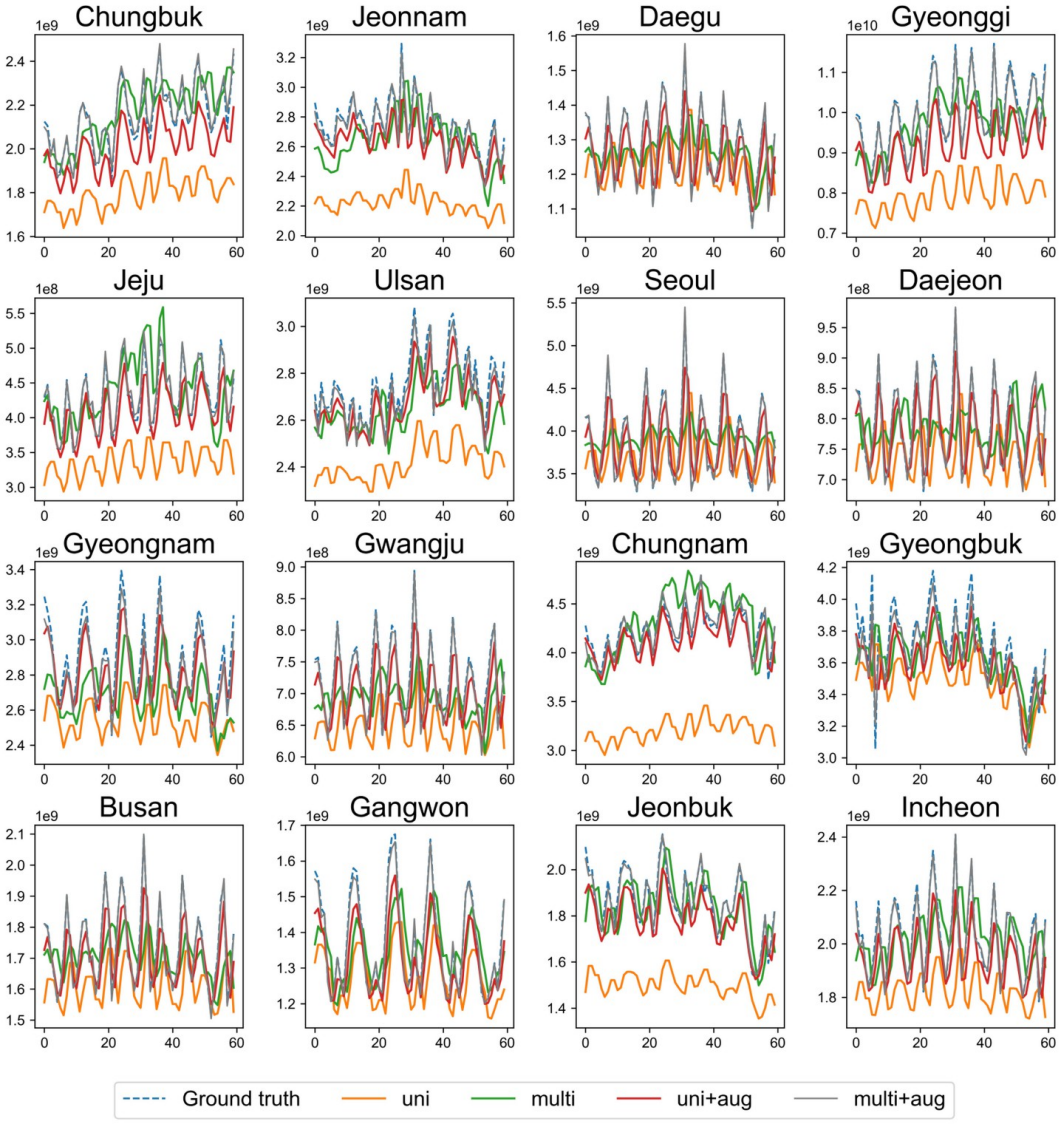
State-level forecasting performance comparison of CNN-LSTM.

We verified the effect of multivariable augmentation on the performance of the CNN-LSTM model. The state-wise monthly electricity consumption prediction obtained using each method on the test dataset of 60 months is illustrated in Fig 5. The proposed method effectively minimizes the error in all regions compared to other methods, and the multivariable augmented flows overlap almost perfectly with the actual power demand data. Only *Gyeongnam*, *Gyeongbuk*, and *Ulsan* exhibit observable differences between the ground truth and multivariable augmentation. Table 4 summarizes the evaluation results of the CNN-LSTM model for 16 regions using different data processing methods. The proposed augmented multivariable model is observed to yield useful results in all states, exhibiting the lowest RMSE and MAPE scores.

**Table 4:** Evaluation of forecasting performance of CNN-LSTM.

Regions	RMSE				MAPE			
	Univariable	Multivariable	Augmented Univariable	Augmented Multivariable	Univariable	Multivariable	Augmented Univariable	Augmented Multivariable
**Busan**	0.2345	0.1722	0.0853	**0.0132**	0.2453	0.2034	0.0935	**0.0154**
**Chungbuk**	0.3649	0.1252	0.1418	**0.0253**	0.3276	0.1038	0.1262	**0.0193**
**Chungnam**	0.3738	0.1108	0.0422	**0.0291**	0.3767	0.0970	0.0373	**0.0235**
**Daegu**	0.2456	0.2053	0.0982	**0.0168**	0.2937	0.2821	0.1193	**0.0216**
**Daejeon**	0.2543	0.2082	0.0782	**0.0157**	0.2638	0.2496	0.0862	**0.0179**
**Gangwon**	0.2038	0.1509	0.0957	**0.0185**	0.2107	0.1872	0.0981	**0.0212**
**Gwangju**	0.2817	0.2014	0.0988	**0.0187**	0.2535	0.1959	0.0939	**0.0191**
**Gyeongbuk**	0.1820	0.1416	0.0874	**0.0496**	0.2151	0.1750	0.1037	**0.0551**
**Gyeonggi**	0.4121	0.1662	0.1525	**0.0201**	0.3754	0.1415	0.1324	**0.0173**
**Gyeongnam**	0.2599	0.1883	0.0735	**0.0403**	0.2870	0.1983	0.0782	**0.0419**
**Incheon**	0.3165	0.1922	0.1141	**0.0209**	0.3168	0.2188	0.1133	**0.0213**
**Jeju**	0.4632	0.2161	0.1210	**0.0273**	0.3834	0.1650	0.0940	**0.0210**
**Jeonbuk**	0.3420	0.1183	0.0799	**0.0232**	0.4185	0.1327	0.0858	**0.0253**
**Jeonnam**	0.3344	0.1368	0.0744	**0.0247**	0.3849	0.1360	0.0744	**0.0248**
**Seoul**	0.2959	0.2519	0.1223	**0.0170**	0.4683	0.4666	0.1890	**0.0274**
**Ulsan**	0.3446	0.1580	0.0828	**0.0379**	0.2837	0.1085	0.0628	**0.0290**

### Ablation studies

**Fig 6:**
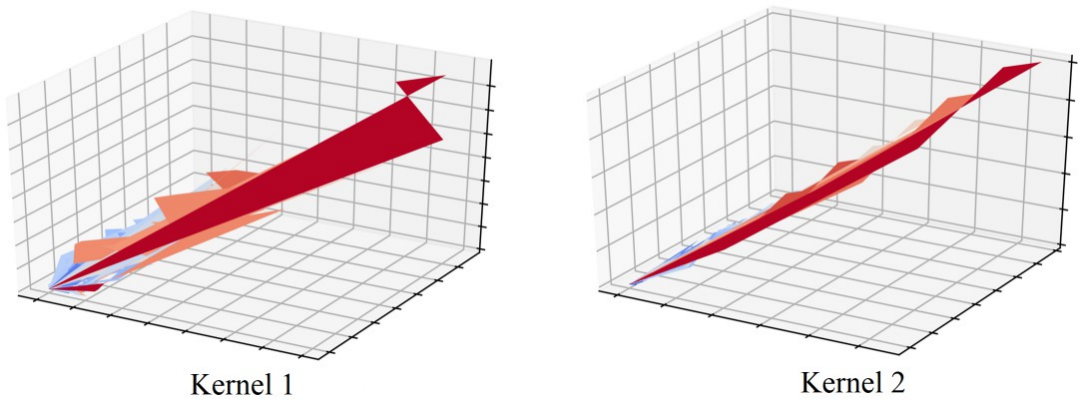
Kernel output comparison of 1D CNN layers.

**Fig 7:**
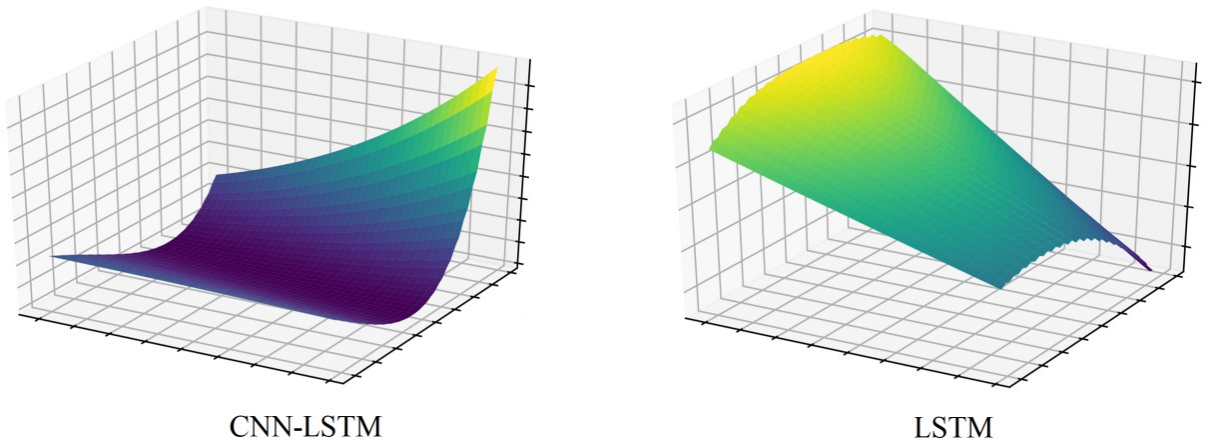
Comparison of loss landscapes of LSTM and CNN-LSTM.

To establish the superiority of the proposed model, two experiments are conducted as ablation studies. First, following the 1D CNN kernel visualization technique presented in [7, 31], Fig 6 illustrates the noise-reducing power of each 1D CNN layer. The intermediate output obtained from the second convolution network is observed to be less spiky than the first kernel. This is because each CNN filter reduces the noise of the input dataset. This selectivity over input information also allows cost-effective computation and is, hence, useful for mid-term time series forecasting. The illustration aids the analysis of the number of layers that benefit from smoothing the noise of raw data and selectively extracting important information. Moreover, the loss landscapes of LSTM and the proposed model are compared in Fig 7. In [32], the loss landscape was introduced as a method to represent loss convergence of a model throughout the training process. From the figure, it is evident that the proposed CNN-LSTM hybrid model converges the loss to the minima satisfactorily, and the graph is smooth and concave, indicating facile training. On the other hand, the LSTM-only model exhibits convex loss surfaces and the minima are not visible, indicating that the training of the model is more difficult. This proves that the inclusion of 1D CNN layers before the LSTM layer simplifies training.

## Discussion

Electricity is the backbone of modern society. As such, the accurate prediction of electricity demand and consumption is more significant now than ever before. However, many factors complicate the forecasting accuracy of energy consumption, necessitating the development of advanced forecasting models. In this paper, we presented a new hybrid CNN-LSTM multivariable EEC forecasting techniques that integrates the advantages of CNNs and LSTMs. To incorporate economic insights into energy usage, the proposed technique was expanded using import/export values. The proposed approach enables the prediction of regional monthly electricity consumption. We tested the proposed techniques (components and algorithm) using baseline techniques on univariable historical energy consumption data and compared them to conventional deep learning models. Comprehensive experiment results proved that the proposed technique extracts useful information between international trade values and electricity usage, thereby improving the accuracy of national/regional predictions. We also expanded the proposed technique to consider data interpolation. The proposed mechanism can be further improved using data augmentation.

## Conclusions

In this study, we investigated the effects of CNN-LSTM on augmented multivariable time series datasets. We concluded that dimension reduction using the pooling layer of 1D CNN reduces noise and thereby reduces the RMSE and MAPE scores. The LSTM layer was also observed to be well suited to process time series data as it receives inputs for each time step. Extensive experiments and ablation studies were performed, establishing the benefits afforded by the proposed CNN-LSTM architecture paired with multivariable augmentation to provincial time series forecasting for EEC.
